# Fear, helplessness, and horror—if it does not stop: reflections on the evolving concept of impact of trauma

**DOI:** 10.3402/ejpt.v6.27634

**Published:** 2015-04-08

**Authors:** Eric Vermetten

**Affiliations:** 1 Department of Psychiatry, Leiden University Medical Centrum, Leiden, The Netherlands; 2 Department of Defense, Military Mental Health Research, Utrecht, The Netherlands; 3 Arq Psychotrauma Expert Group, Diemen, The Netherlands

War psychiatry has served as the backbone of our current understanding of the impact of psychotrauma. The First World War confronted the world with an invalidating phenotype that has since then seen itself represented with different names, of which shell shock is probably best known. Since then wars have provided us a moral imperative to better understand and prevent the impact of combat (see special issue EJPT, 2014; Yehuda, Vermetten, & McFarlane, 2014). Wars, but also events such as armed robbery, rape, accidents, and natural disasters, can confront an individual with danger to life, injury to oneself or another person, or in the worst case death. We categorize so-called “near death” or “serious injury” experiences as traumatic stressors. For some, they may be associated with intense fear, horror, and helplessness and can cause serious lasting dysregulations of daily life. It can cause a dysfunctional repetitive cycle from which it is not easy to “snap out of” (Bremner & Reed, 2014). For some, life is no longer the same, and one's life narrative cannot stop gravitating toward the traumatic events. The fear, helplessness, and horror as well as of symptoms, nightmares, flashbacks, avoidance, hopelessness, guilt, irritability, memory, and concentration difficulties and sleep problems can serve as clinical signifiers. It lasted until 1980 before and cause the an phenotype twas hat included exists since 1980 in the third edition of the Diagnostic Statistical Manualpsychiatric classification as post-traumatic stress disorder (PTSD) (Van der Kolk, 2014; Vermetten, Kleber, & Van der Hart, 2012).

Since then, three decades of research have passed (Vermetten & Lanius, 2012), in which the number of studies on PTSD have exponentially increased. The studies have moved from identification to validation, treatment, and also prediction, and shifted from tertiary to primary prevention and resilience enhancement. But with the increasing number of studies, the number of questions has also increased. Most importantly, what do we know now what we did not know then? Has our knowledge led to a greater understanding by mental health professionals? Has it been incorporated in the educational curricula of universities? Has the knowledge led to new treatment opportunities? Can you “heal” or “cure” PTSD?

A century ago, the term “shell shock,” a medical label, was introduced to represent the ones with severe disturbances due to exposure to shells of the war. Yet, there was no good concept for the disorder, because it occurred also in soldiers that had not been exposed to shells. The Kriegstraumatische Zitterneurose, as Germans labeled it, left room for controversy as this did not uniquely focus on external causes. A world war later, with devastating experiences of the Holocaust and names like “physioneurosis” and “KZ syndrome,” we became even more aware of the unique attribution of the clinical phenomenology to traumatic stress, and moved away from “mental breakdown” or “moral weakness” (Weisaeth, 2002). Yet it took many more years to recognize the full impact of traumatic events as precipitating events and find a discourse within neurosciences with notions of, for example, allostasis (McEwen, 1988) and resilience (Bonanno, 2004). Pivotal was the Post–Vietnam War movement that helped embrace the lasting impact of trauma exposure in DSMIII. Salience did not lead to simplicity. Despite preservation of the name PTSD and moving away from the category of anxiety disorders in DSM5, it has not become easier, or to say the least “simple.”

To list a few challenges:

- The current DSM5 lists 23 symptoms that need to be present in a variety of combinations to qualify someone as suffering from PTSD, contributing to a heterogeneity of the disorder.
- New questions emerge about the complexity of the disorder, for example, the dissociative element, and discussions of “simple” vs “complex”.
- The majority of people exposed to trauma will not be clinically affected. There are important notions about resilience, what drives resilience?
- We revisit questions that drove the post war period of the First World War about stigma, moral weakness, or injury, and the discrimination of PTSD with “visible” disorders such as mild traumatic brain injury (mTBI).
- The PTSD of today is a grimsy disorder, does not have a clear incubation time, and varies in its presentation in relation to the time of onset after exposure.
- It is a disorder that for diagnosis is purely based on self-disclosure, self-observation. We seem to completely go beyond behavioral observations as important assessment elements, and do not use hetero-anamnestic information as a clinical or critical source of information.

Perhaps most important of all, despite promising new findings, there is also still no “biological qualifier,” which serves to determine who is more or less likely to get it, if exposed. No “mental Cooper test” exists, that can help us to select who is susceptible to the disorder. We invest many resources since it is felt that this prediction will not only be possible but also desperately wanted. Militaries and other uniformed services are dying to know how to implement this in selection procedures (Yehuda et al., 2014), saving young men and women from unnecessary suffering. How far are we from first implementation of biomarkers in selection procedures? Do we see breakthroughs emerging to support this? In our fourth decade of research since 1980 with rapid developments in (epi)genetics, optogenetics, and novel imaging methods, this may be not that far down the road (Vermetten, Baker, & Yehuda, 2015; Vermetten, Zohar, & Krugers, 2014).

Psychological trauma has become an iconic element in our society. Starting little over 100 years ago, psychotraumology is like a fast moving train, with currently more than 2,500 scientific publications per year on the topic of PTSD. The field is presenting itself to a highly modernized world, where stakes are high and education of a discourse is highly needed, because trauma is not likely to disappear. The artificial dichotomy between vulnerability and resilience is challenged by increasing violence, natural, and man-made disasters, and by participation in small conflicts and big-scaled wars. Murray and Lopez predicted in the Lancet in 1997 that in 2020 war and violence would be ranked in the top 10 of “disease burden” (Murray & Lopez, 1997). If they are right, this puts a burden on the society. Do we habituate? Or sensitize? Yet in some countries, academia is hesitant to embrace or acknowledge the impact for the small group that is suffering, or one is not thinking yet in terms of healing and supportive communities or societies. Maybe the most important lesson we learned over the last years is to acknowledge trauma, identify the ones affected in need, and prevent the “engraving” of the impact of the stress early. In order to do so, we must be able to identify the persons that are biologically vulnerable representing a vulnerable phenotype, in order to justify early treatment, or monitor the developmental course in the aftermath of trauma exposure recommendation and facilitate his or her empowerment.
